# Fabrication of Long Period Gratings by Periodically Removing the Coating of Cladding-Etched Single Mode Optical Fiber Towards Optical Fiber Sensor Development

**DOI:** 10.3390/s18061866

**Published:** 2018-06-07

**Authors:** Joaquin Ascorbe, Jesus M. Corres, Ignacio del Villar, Ignacio R. Matias

**Affiliations:** 1 Department of Electrical, Electronic and Communication Engineering, Public University of Navarra, 31006 Pamplona, Spain; jmcorres@unavarra.es (J.M.C.); ignacio.delvillar@unavarra.es (I.d.V.); natxo@unavarra.es (I.R.M.); 2 Institute of Smart Cities, Public University of Navarra, 31006 Pamplona, Spain

**Keywords:** long period fiber gratings, laser ablation, micromachined, optical fiber sensors, thin-film coatings, refractometer

## Abstract

Here, we present a novel method to fabricate long period gratings using standard single mode optical fibers (SMF). These optical devices were fabricated in a three-step process, which consisted of etching the SMF, then coating it with a thin-film and, the final step, which involved removing sections of the coating periodically by laser ablation. Tin dioxide was chosen as the material for this study and it was sputtered using a pulsed DC sputtering system. Theoretical simulations were performed in order to select the appropriate parameters for the experiments. The responses of two different devices to different external refractive indices was studied, and the maximum sensitivity obtained was 6430 nm/RIU for external refractive indices ranging from 1.37 to 1.39.

## 1. Introduction

Long period gratings (LPGs) have been extensively studied for the last few decades [1]. LPGs are structures where the periodic modulation of the refractive index (RI) along the length of the optical fiber leads to light coupling between the core mode and co-propagating cladding modes at specific resonance wavelengths [2]. Knowing that the attenuation bands in the optical spectrum are generated by the coupling of the fundamental mode to cladding modes of the LPG structure, there is a significant dependence between the properties of the external medium and the position of the attenuation bands [3].

The first application found for LPGs was as rejection band filters [1]. However, applications as optical fiber sensors were developed soon afterwards. This was possible thanks to the interaction of the cladding modes with the external medium [4,5,6]. Based on this property, LPGs are inherently sensitive to physical parameters, such as temperature [7,8], strain [9], surrounding medium RI (SMRI) [10], and bending radius [11]. However, they can be made sensitive to chemical parameters if they are coated with an appropriate material [12,13,14]. In fact, LPG-based sensors for humidity [15,16,17], pH measurements [18,19], volatile organic compounds [20], chemical species detection [21], gas detection [22], or even biological sensors [13,23] have been fabricated. 

Two different aspects should be taken into account when developing an optical fiber sensor based on LPGs: The fabrication process and the sensitivity.

Regarding the fabrication, the most well-known method for inscription of LPGs is a core RI change induced by UV laser radiation in standard communication fibers [1]. However, several other mechanisms have been exploited to obtain this optical structure: CO_2_ [24] and IR femtosecond laser radiation [25], ion beam irradiation [26], etching [27,28], mechanically induced gratings [29], and electric arc discharges [30]. Besides, different kinds of fibers have been used for the development of this optical structure, such as standard single mode optical fibers (SMF28) [6], hydrogen loaded fibers [31,32], and Ge doped [33] and B/Ge co-doped fibers [30]. 

Other methods, based on lithography or wet-etching processes, have recently been used for the fabrication of the so-called corrugated LPGs [27,28,34,35,36,37,38]. During these processes, sections with different effective indices (*n_eff_*) were obtained by periodically decreasing the cladding diameter. The sensitivity of this optical structure towards different parameters [38]—strain, torsion, and bending—has been studied, obtaining some promising results. A method based on coating the multimode section of a tapered SMF through an amplitude mask has also been demonstrated for LPG development [39]. Several modes can be guided through the tapered section of an SMF, making it more difficult to predict coupling interactions between these different modes and, consequently, to optimize that optical platform.

Regarding the sensitivity of LPGs, great efforts have recently been made to increase them. This goal can be achieved using different techniques, such as adding a coating with high RI [40,41], working in the dispersion turning point (DTP) [42,43,44,45,46,47], or reducing the diameter of the cladding by etching [48] or tapering the fiber [49]. 

The method proposed here consists of the periodic laser ablation of a coating previously deposited on a chemically etched SMF, which will keep single mode propagation because the core of the fiber remains unaffected. The optical device developed here combines some of the characteristics that make LPGs more sensitive, such as an etched optical fiber and further deposition of a thin-film coating, which will be periodically removed later. The fabrication process offers some advantages over other methods, such as lithography. There are more available materials, the number of fabrication steps is lower compared to corrugated LPGs [35], it can be developed in standard SMFs, and physical parameters or chemical species affecting the coating will modify the grating itself, either by changing its refractive index or by changing its size. As a demonstration of this novel optical structure, the behavior as a refractometer was studied.

## 2. Fabrication Process

**Figure 1:**
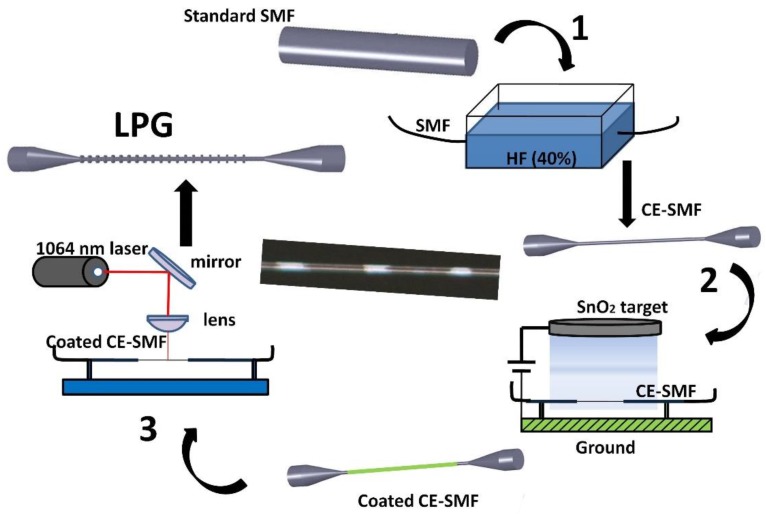
Scheme of the fabrication process and photo of the final device (device B). Step 1: Chemical etching of the SMF. Step 2: Coating the CE-SMF by sputtering. Step 3: Inscription of the grating by laser ablation.

The fabrication process of the proposed device is described in Figure 1. This optical device was fabricated in a three-step process, which consists of etching the SMF, then coating it with a thin-film and, the final step, which involves removing sections of the coating periodically by laser ablation. For the chemical etching, hydrofluoric acid (HF) diluted at 40% (Sigma-Aldrich, St. Louis, MO, USA) was used. The SMF (SMF28e with 8/125 μm core/cladding diameter purchased from Telnet RI) was immersed in the HF (previously 50 mm of the buffer was removed). It was important to remove the buffer in a longer section than the one that was immersed in HF to avoid the fiber becoming brittle. When the overall diameter reached 12 μm, the etched SMF was washed with deionized water to remove all the HF. The diameter of the fiber was measured using the software included in the microscope (Leica DMC 2900, Leica Microsystems, Wetzlar, Germany). This measurement was performed before gluing the fiber to its holder. If the diameter was not the desired one, the fiber was immersed again in HF until the diameter met the requirements. Then, it was attached to a U-holder to keep the fiber straight and, hence, to avoid any bending artifacts. 

Once the fiber reached its final diameter, a tin dioxide thin film was deposited on the cladding-etched SMF (CE-SMF). Tin dioxide was sputtered by means of a pulsed DC sputtering system (Nadetech Inc., Pamplona, Spain). SnO_2_ was selected between different transparent conductive oxides (TCO) because of its optical properties, which meet the two main characteristics desired: a high refractive index and a low extinction coefficient (these are the requirements to obtain a sensitive device whose attenuation band exhibits a low spectral width). Pulsed DC sputtering technique has been selected because of its high repeatability, high thickness control, and because it is a fast and reliable method. The sputtering process was performed at a vacuum chamber pressure of 2 × 10^−2^ mbar, using argon as the process gas and with a sputtering current of 180 mA. The coating was deposited homogeneously around the fiber, enabling the obtainment of a polarization independent device. 

Two devices with different coating thickness were developed. The first one, device A, was coated with 55 nm of tin dioxide, whereas the coating thickness of device B was 65 nm. The greater thickness of device B allows for reducing the number of periods required to generate the resonances in the optical spectrum: 120 for device A and 100 for device B. Consequently, the grating length can be reduced at the same time. The thickness was measured using a quartz crystal microbalance (QCM). 

Then the coated CE-SMF was placed under the lens of the laser (PEDB-100, Perfect Laser, Wuhan, China). This laser emits at 1064 nm with a maximum power of 20 W and is pulsed at a frequency of 20 kHz. A digital galvanometer scanning head positions the laser beam. The beam power, the repetition rate, and the scanning velocity can be adjusted by software, which allows for an accurate control of the laser direct writing (LDW). Here, the laser was programmed to periodically evaporate sections of the coating. The transmitted spectrum was monitored after each period was written. The transmitted optical spectra were normalized with respect to the one provided by a SLED light source (HP-83437A, Agilent, Santa Clara, CA, USA), and they were acquired by an optical spectrum analyzer (HP-86142A, Agilent, Santa Clara, CA, USA). Monitoring the transmitted optical spectrum was useful for checking that the laser was operating correctly. Device A and device B have the same overall diameter of 12 µm. The period was adjusted to 200 µm for device A and 160 µm for device B, in order to be able to measure changes in different RI ranges, as will be seen later.

## 3. Theoretical Analysis

**Figure 2:**
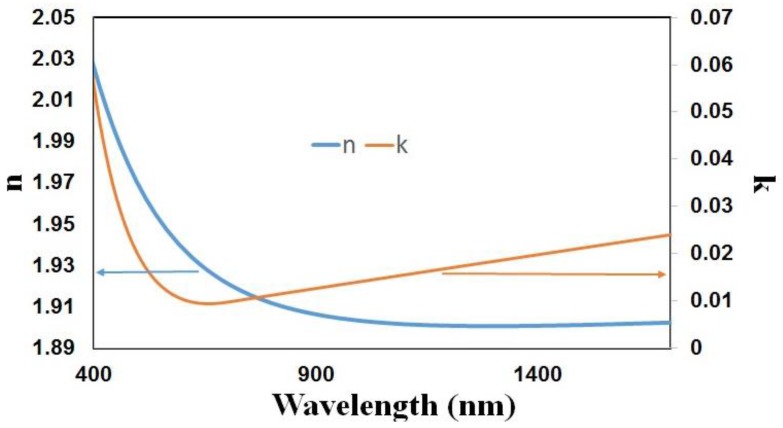
Refractive index of the tin dioxide thin film acquired with ellipsometer Uvisel-2 (Horiba, Kioto, Japan).

This section presents a theoretical analysis that was performed for the parameters of the structure presented in Section 2. The SMRI was set to 1.321 [50] to simulate water as the surrounding medium. FIMMWAVE^®^ was used for the numerical analysis of the structure. The RI of the optical fiber cladding, made of fused silica, was estimated with the Sellmeier equation [30]. The optical fiber core RI for the simulations was obtained, according to the specifications from Corning^®^, by increasing the RI of the cladding by 0.36%. The material used for the simulations as well as for the experiments is tin dioxide, whose RI was measured by ellipsometry (Uvisel-2 from HORIBA, Kioto, Japan), and is shown in Figure 2.

### 3.1. Influence of the Diameter of the Fiber

**Figure 3:**
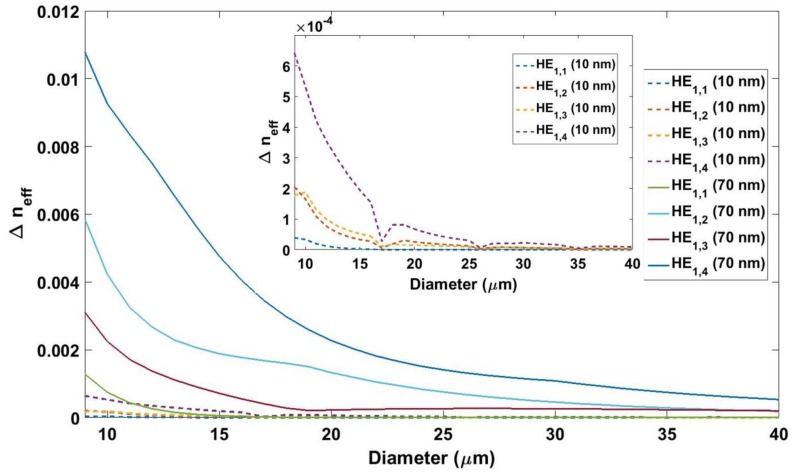
Difference between the *n_eff_* of the coated sections and the uncoated section as a function of the diameter for thicknesses of 10 and 70 nm. The fundamental mode and the three first cladding modes were analyzed. Thickness of 70 nm is represented as continuous lines, and thickness of 10 nm is represented as dashed lines, which is also depicted in the inset.

In the first place, the influence of the overall diameter of the fiber was analyzed. For this purpose, the *n_eff_* of the fundamental mode, and of some cladding modes, was calculated for each wavelength from 1200 to 1700 nm for different thicknesses of tin dioxide and for different overall diameters of the fiber. In addition, the *n_eff_* of the modes was calculated under the same conditions, except for the fact that this time no tin dioxide coating was included (i.e., the profile of the uncoated section was analyzed). In order to understand the results of the simulations, a specific case is presented in Figure 3. The *n_eff_* of sections coated with 10 and 70 nm of SnO_2,_ and the *n_eff_* of the uncoated section for fibers of different diameters, were calculated. Then, the difference between the *n_eff_* of the coated sections and the uncoated section was plotted as a function of the diameter for thicknesses of 10 and 70 nm, in order to see the effect of decreasing the diameter.

The change in *n_eff_* increased for higher order modes, but the main conclusion is that the scale of the change for the 9 µm fiber was 50 times higher than in the fiber of diameter 40 µm. Moreover, unlike in the 40 µm fiber, in the 9 µm fiber there was a change in the *n_eff_* of the fundamental mode. This higher perturbation suggests that a low diameter fiber must be used, as it will be the case in Section 4. 

**Figure 4:**
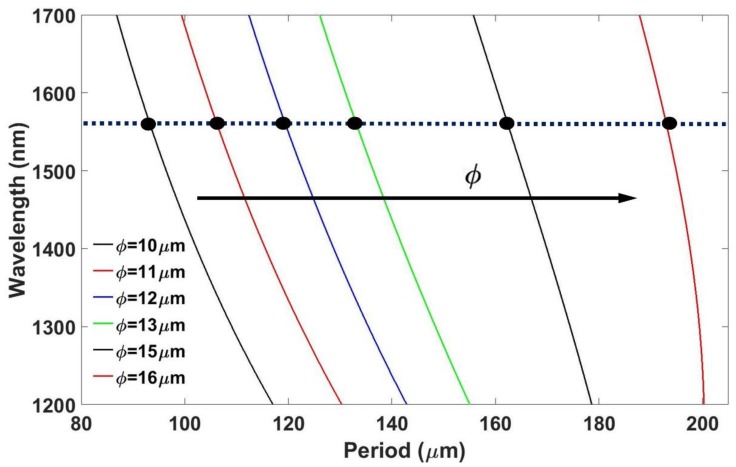
Resonance wavelength as a function of the grating period for 50 nm tin dioxide coated fibers of different diameters corresponding to the mode $HE_{1,4}^{2}$. The horizontal line and the black spots represent the working points for further analysis.

These changes in the *n_eff_* of the core and the cladding modes play a role in the coupling between the core mode and each cladding mode (i.e., on the generation of resonances in the transmission spectrum). The simplest way to calculate the position in the optical spectrum of these resonances for each period is the well-known phase matching condition [51]:

(1) λ=[neff(λ)−ncladi]ΛN

where *n_eff_* (λ) is the effective RI of the propagating core mode at wavelength *λ*, *n^i^_clad_* (*λ*) is the effective RI of the *i*^th^ cladding mode, Λ is the period of the LPG, and *N* is the diffraction order. Taking into account this equation and the data obtained previously, it was possible to calculate the wavelength of the resonance as a function of the grating period. The lines satisfying the Bragg condition corresponding to the mode $HE_{1,4}^{2}\;$ for a thin-film of 50 nm coated onto a CE-SMF of different diameters are shown in Figure 4.

In Figure 4, the wavelength of the resonance is represented as a function of the grating period for diameters increasing from 10 to 16 µm. For analyzing the influence on the sensitivity of the diameter, all the other parameters needed to remain unaffected. Therefore, the thickness of the coating, the mode that is going to be coupled, and the initial wavelength of the resonance were kept constant. Then, in order to obtain the resonance located at the same wavelength and to couple the same cladding mode, it became necessary to change the grating period. 

**Figure 5:**
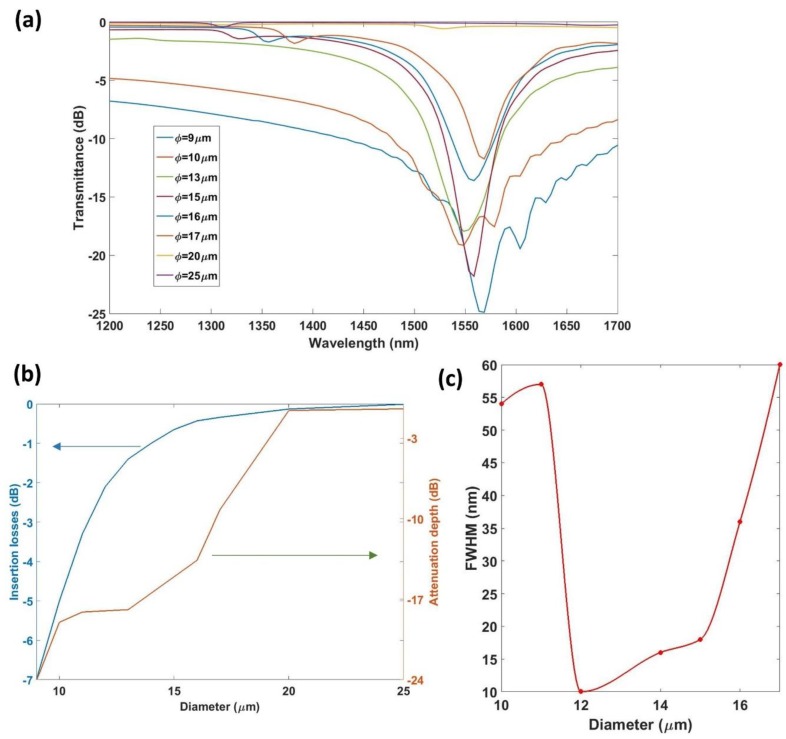
(**a**) Simulated spectra for an LPG written onto a 50 nm SnO_2_ coating for different overall diameters of the cladding-etched fiber; (**b**) insertion losses and attenuation depth; (**c**) FWHM as a function of the diameter.

Following the procedure to select the grating period schematized in Figure 4, the influence of the diameter on the attenuation band, as well as the influence of the diameter on the sensitivity, were analyzed. Figure 5 shows the influence of the diameter on the transmitted spectrum obtained, as well as on the characteristics of the attenuation band. The data were obtained by simulating an LPG with 120 periods written onto a 50 nm tin dioxide thin-film. The number of periods was chosen by comparison with conventional LPGs and because it offers a reasonable final length of the device. The grating period was calculated to couple the $HE_{1,4}^{2}$ mode, as indicated in Figure 4.

Figure 5a shows the transmitted spectra for different diameters, whereas Figure 5b shows the insertion losses and the attenuation depth of the resonance as a function of the diameter. Finally, in Figure 5c, the full width at half maximum (FWHM) is plotted against the diameter.

**Figure 6:**
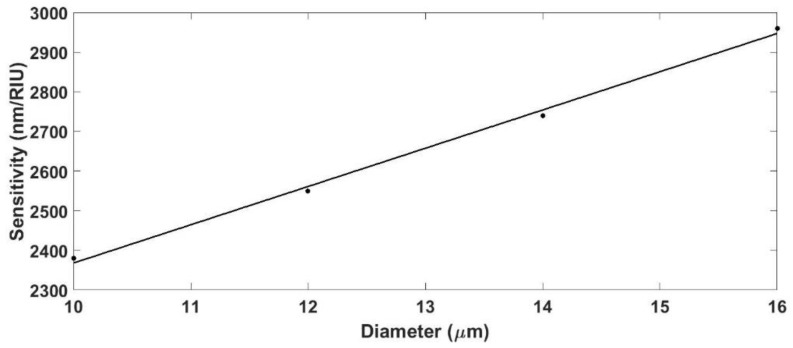
Sensitivity of an LPG written onto a 50 nm thin-film coated cladding-etched single mode optical fiber as a function of the diameter.

From Figure 5, it can be concluded that diameters greater than 18 µm do not allow for a resonance with coupling strength greater than 3 dB for the number of periods studied. There is a range of diameters, between 12 and 15 µm, which provide the smallest FWHM. In addition, a study of the influence of the diameter on the sensitivity was also performed and the obtained results are represented in Figure 6. It is important to remark that the grating period was selected to always work with the same mode coupled (mode $HE_{1,4}^{2}$ in this case), which was selected to also obtain the resonance at the same wavelength every time, in order to avoid the dependence of the sensitivity on the wavelength. The SMRI was swept from 1.321 to 1.351, and the diameter was swept from 10 to 16 µm, while the thickness of the coating was fixed at 50 nm.

The sensitivity increased following a linear behavior as the diameter increases. However, the FWHM of the resonance also increased with the diameter. For the following analysis and for the experimental study, the diameter was fixed to 12 µm as a compromise between sensitivity and spectral width. This diameter enables a significant modification of the *n_eff_* of the core and the cladding modes by deposition of a thin-film, providing an attenuation band that can be easily monitored and distinguished. 

### 3.2. Influence of the Thickness of the Coating

**Figure 7:**
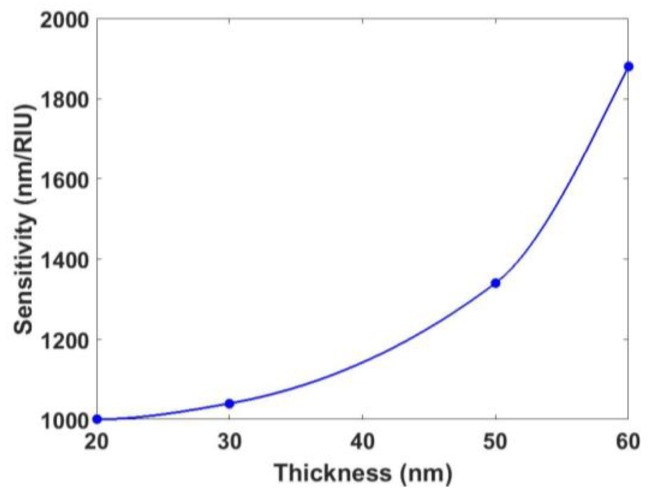
Sensitivity of an LPG written onto a 12 µm CE-SMF as a function of the thickness of the supporting layer.

The next parameter that was analyzed was the thickness of the thin-film where the grating was written. Following the same scheme as in the previous section, the *n_eff_* of both the core and the cladding modes was calculated as a function of thickness and for a fixed diameter of 12 µm. The SMRI was swept from 1.321 to 1.351, and the thickness was swept from 20 to 60 nm. The grating period was fixed in such way that it worked with the resonance associated with the mode $HE_{1,3}^{1}$. Figure 7 shows the sensitivity as a function of the coating thickness. 

Figure 7 shows the influence of the thickness of the coating in the sensitivity of the device. Here, the relationship between thickness and sensitivity was stronger than the relationship between diameter and sensitivity. For the thinnest coating (20 nm), the wavelength shift of the resonance followed an almost linear behavior with a sensitivity of 1000 nm/RIU. However, as the thickness increases, the behavior of the device evolved towards a quadratic behavior with a maximum sensitivity of 1900 nm/RIU (for a thin-film of 60 nm). Nonetheless, it must be highlighted that the maximum thickness was limited by the absorption of the material, which prevented increasing the thickness indefinitely.

### 3.3. Influence of the Grating Period

**Figure 8:**
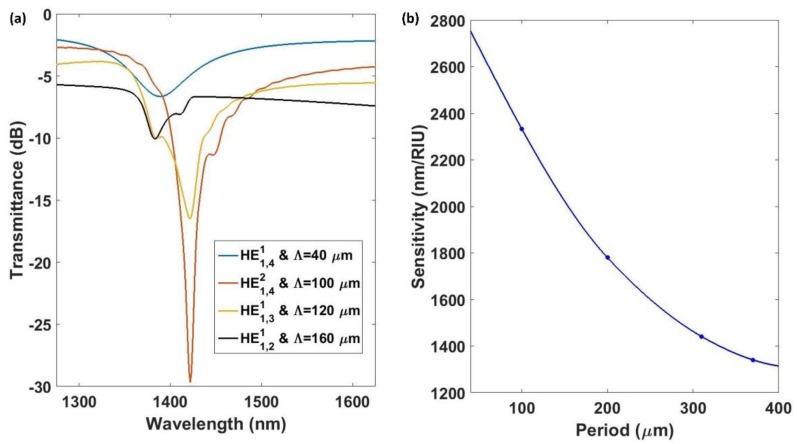
(**a**) Transmission spectra for the first four resonances; (**b**) sensitivity of an LPG written onto a 12 µm CE-SMF as a function of the grating period.

In conventional LPGs, the sensitivity is dependent on the grating period. For this section, the dependence was analyzed. To this purpose, a device of 12 µm of diameter with a coating of 40 nm was studied. The SMRI was swept from 1.321 to 1.351, and the grating period was swept, in a discrete way, from 50 µm to 370 µm. The sensitivity as a function of the grating period, as well as the transmission spectra, are shown in Figure 8.

It was observed that each coupled mode generated a resonance with different properties. Resonances associated with the mode $HE_{1,4}^{1}$ were more sensitive than the others, but the FWHM was the greatest (120 nm). However, the next resonances that appeared, which were associated with the modes $HE_{1,4}^{2},\;HE_{1,3}^{1},\;$ and $HE_{1,2}^{1},\;$ presented a FWHM of 10, 20, and 32 nm, respectively. Therefore, it seems to be preferable to work with these modes. Although they lead to lower sensitivities, this drawback will be compensated for with a greater resolution.

## 4. Experimental Results

Taking into account the simulated results, it was possible to select the appropriate parameters for the fabrication of this kind of device. In this section, the experimental results obtained for two different devices are explained. The final chosen diameter of 12 µm was chosen as a compromise between sensitivity, spectral characteristics of the attenuation band, and the final size of the device. These two devices have been developed using tin dioxide for both of them, but the LPGs have coatings with different thicknesses and different grating periods. Knowing that the sensitivity increases for thicker coatings and shorter grating periods, we compared two devices: one with a thicker coating and a shorter grating period than the other one.

### 4.1. Device A

**Figure 9:**
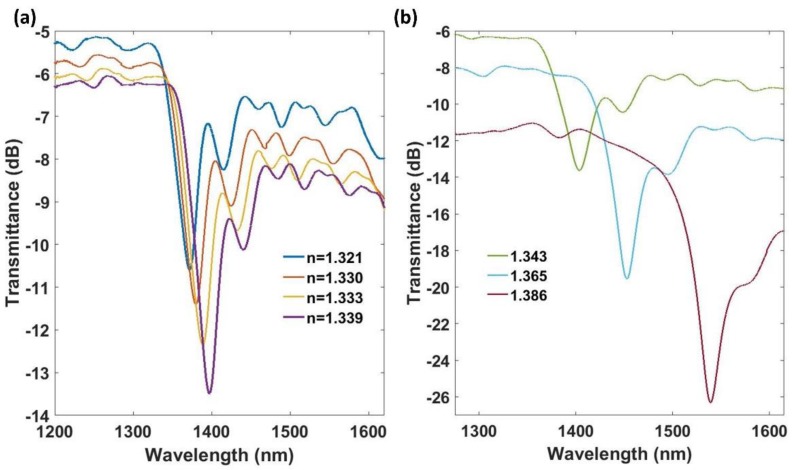
Experimental transmitted spectra of device A for SMRIs ranging (**a**) from 1.321 to 1.339, and (**b**) from 1.343 to 1.386.

Device A was generated by coating an etched fiber of 12 µm in diameter with a thin-film of tin dioxide of 55 nm. The required grating period was calculated using Equation 1, and a grating period of 200 µm was selected for this first device. Then, the coated fiber was placed under the laser and the LPG was generated. Device A, with a nanocoating of period 200 µm, allowed for observing the resonance corresponding to the mode $HE_{1,3}^{2}$ at 1370 nm when it was immersed in water. The experimental dependence of the resonance wavelength of this optical structure on the SMRI was studied. To achieve this purpose, the LPG was immersed into different water-glycerin solutions. The RI of these solutions was first measured with a commercial refractometer (Mettler Toledo 30GS, Columbus, OH, USA). The transmission spectra for SMRI ranging from 1.321 to 1.386 are shown in Figure 9.

The experimental results presented in Figure 8 show that the resonance redshifts as the SMRI increases and that there is also a continuous increase of the attenuation depth. The resonance of conventional LPGs blueshift as the SMRI increases for grating periods above the dispersion turning point, whereas for grating periods below the dispersion turning point, there is a redshift. This behavior is related to the shape of the lines satisfying the Bragg condition. If they present a positive slope, there is a blueshift, whereas if they show a negative slope, there is a redshift. In addition, it has been checked recently that as the diameter of the optical fiber is reduced, the dispersion turning point is shifted to lower grating periods. Consequently, the grating period range where there is a redshift increases [46]. In the structure analyzed in this work, the diameter was very low and the grating period was also short. As a result, a redshift in the resonances was observed when the SMRI was increased. The attenuation band shifted 167 nm, providing a maximum sensitivity of 4000 nm/RIU in the 1.343–1.386 range. It was observed that the wavelength shift was non-linear. The FWHM of the resonance was 40 nm at 1.321 and 20 nm at 1.386.

### 4.2. Device B

**Figure 10:**
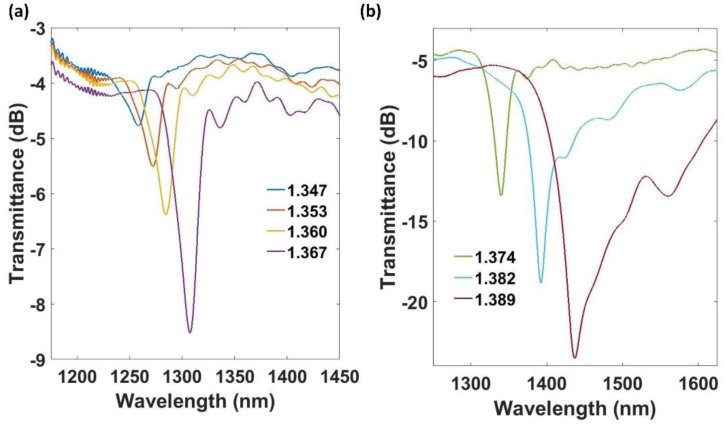
Experimental transmitted spectra of device B (40 nm) for SMRI ranging (**a**) from 1.347 to 1.367, and (**b**) from 1.374 to 1.389.

Another device fabricated with different parameters, called device B, was analyzed. As it was indicated in Section 3, the thickness of the thin-film was increased for this device in order to improve the sensitivity of the final device. Then, the thickness of the coating for device B was increased to 65 nm. Again, simulations became crucial to know the appropriate grating period. Now, in order to have the same mode coupled as the one in device A, a period of 160 µm was selected. According to LPG theory, shorter periods will lead to higher sensitivity towards the external refractive index [43]. Device B, with a nanocoating of period 160 µm, allowed for observing the resonance corresponding to the mode $HE_{1,3}^{2}$ at 1280 nm when it was immersed in a medium with a SMRI of 1.347. Using these parameters, the evolution of the transmitted optical spectrum of device B was experimentally studied for different RIs (see Figure 10). The attenuation band redshifted as the SMRI increased, and there was also a continuous increasing power loss as the SMRI increased. This increase of the attenuation was smaller than that one observed for device A, due to the lower number of periods. The attenuation band shifted 179 nm, providing a maximum sensitivity of 6430 nm/RIU in the 1.374–1.389 range. The FWHM of the resonance obtained was 30 nm at 1.367 and it diminished to 20 nm at 1.389.

### 4.3. Comparison between Theoretical and Experimental Results

**Figure 11:**
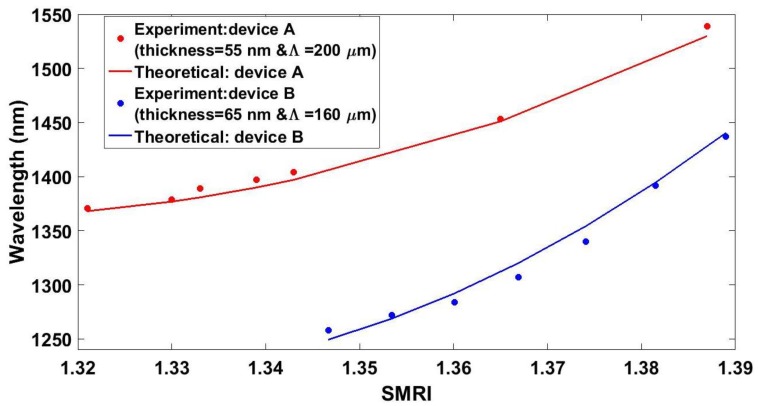
Wavelength of the resonance band as a function of the surrounding medium’s refractive index.

Figure 11 shows the wavelength of the resonance as a function of the SMRI. A good agreement between experimental results and theoretical results was obtained, which indicates that the model presented in Section 2 was useful for the design of this optical device. In addition, the wavelength shift was non-linear, as is the common behavior for an LPG [47]. The sensitivity for refractive indices between 1.343 and 1.386 was 4000 nm/RIU for device A. Device B provided a sensitivity of 6430 nm/RIU for SMRIs between 1.374 and 1.389, which were larger than those obtained in DTP-tuned LPGs in the telecommunications spectral range without cladding reduction (1309 nm/RIU in Reference [52], 1847 nm/RIU in Reference [53], and 944 nm/RIU in Reference [54]), and competes with the value of 8374 nm/RIU obtained with DTP-tuned LPFGs in the telecommunications spectral range with cladding reduction [46]. 

## 5. Conclusions

Here, a novel method to obtain an LPG-based optical structure was developed and the wavelength dependence of the attenuation band on the SMRI was studied. The combination of sputtering and laser ablation provided a method for the development of this kind of optical structure, which can be tuned for working in different conditions (i.e., different SMRIs). The device was developed using standard single mode optical fibers, and the coating material was selected for the development of sensors for different applications. The CE-SMF, which keeps single mode propagation, made this structure highly predictable. Therefore, this kind of device can be easily tuned for each coating material and for working in a specific RI range.

The maximum sensitivity, obtained with device B, was 6430 nm/RIU for SMRIs between 1.374 and 1.389; a value that competes with those ones obtained with LPGs fabricated using other techniques. Since the nanocoating material was responsible for the grating generation, the sensitivity of the device was enhanced with the deposition of other materials with higher refractive index. Moreover, the deposited material was sensitive to a specific parameter to detect. This widens the domain of application of this type of device. In this sense, further research should be done to develop sensors for specific application fields, such as pH sensors, detection of chemical or biological species, magnetic field sensors, etc.
